# Impact of a genomic classifier of metastatic risk on postoperative treatment recommendations for prostate cancer patients: a report from the DECIDE study group

**DOI:** 10.18632/oncotarget.918

**Published:** 2013-09-08

**Authors:** Ketan Badani, Darby J. S. Thompson, Christine Buerki, Elai Davicioni, Jill Garrison, Mercedeh Ghadessi, Anirban P. Mitra, Penelope J. Wood, John Hornberger

**Affiliations:** ^1^ Department of Urology, Columbia University, New York, NY USA; ^2^ EMMES Canada, Burnaby, BC CAN; ^3^ GenomeDx Biosciences Inc., Vancouver, BC CAN; ^4^ Cedar Associates LLC, Menlo Park, CA USA; ^5^ University of Southern California, Los Angeles, CA USA; ^6^ Stanford University, Stanford, CA USA

**Keywords:** MET, gastric cancer, gene amplification, FISH, PCR

## Abstract

**Background:**

Only a minority of prostate cancer patients with adverse pathology and biochemical recurrence (BCR) post radical prostatectomy (RP) experience metastasis and die from prostate cancer. Improved risk prediction models using genomic information may enable clinicians to better weigh the risk of metastasis and the morbidity and costs of treatment in a clinically heterogeneous population.

**Purpose:**

We present a clinical utility study that evaluates the influence on urologist treatment recommendations for patients at risk of metastasis using a genomic-based prediction model (Decipher^TM^).

**Methods:**

A prospective, pre-post design was used to assess urologist treatment recommendations following RP in both the adjuvant (without any evidence of PSA rise) and salvage (BCR) settings. Urologists were presented de-identified pathology reports and genomic classifier (GC) test results for 24 patients from a previously conducted GC validation study in high-risk post-RP men. Participants were fellowship trained, high-volume urologic oncologists (n=21) from 18 US institutions. Treatment recommendations for secondary therapy were made based solely on clinical information (pre-GC) and then with genomic biomarker information (post-GC). This study was approved by an independent IRB.

**Results:**

Treatment recommendations changed from pre-GC to post-GC in 43% of adjuvant, and in 53% of salvage setting case evaluations. In the adjuvant setting, urologists changed their treatment recommendations from treatment (i.e. radiation and/or hormones) to close observation post-GC in 27% of cases. For cases with low GC risk (<3% risk of metastasis), observation was recommended for 79% of the case evaluations post-GC. Consistent trends were observed in the salvage setting.

**Conclusion:**

These results indicate that urologists across a range of practice settings are likely to change treatment decisions when presented with genomic biomarker information following RP. Implementation of genomic risk stratification into routine clinical practice may better direct treatment decision-making post-RP.

## INTRODUCTION

Prostate cancer presents a significant population health burden in the United States. As the most frequently diagnosed cancer among men, almost 240,000 new cases are projected for 2013 [1]. About half of these men will be treated with radical prostatectomy (RP) [2] and while many will achieve a durable cure, up to 50% will present with one or more adverse pathology features such as, seminal vesicle invasion (SVI), extracapsular extension (ECE) or positive surgical margins [3, 4]. Although these patients are considered by guidelines to be at an increased risk for disease progression, only a minority will develop metastatic disease and ultimately die of prostate cancer [5]. Further, while close monitoring with postoperative PSA testing can identify men at risk, the time to biochemical recurrence (BCR) after RP is not predictive for metastatic disease [6]. And, while PSA doubling time (PSAdt) is a good surrogate for aggressive disease, its accurate determination may not be possible in all patients as it requires a certain timeframe during which clinical progression may occur [7].

Treatment recommendations from the National Comprehensive Cancer Network (NCCN) guidelines post-RP include radiation and/or hormone therapy or observation (active surveillance). These guidelines are based in part on results from three independent phase III randomized clinical trials that have demonstrated improvements in BCR-free, metastasis-free and cancer-specific survival in high-risk post-RP men treated with radiation therapy [8-10]. Despite this, deciding on appropriate use of radiation therapy post-RP remains a challenging task. Inappropriate or over-utilization of secondary therapy in this population is of great concern because of the knowledge that most clinically high-risk, post-RP patients will never develop metastasis. Recognizing these factors, guidelines state that “predicting prognosis is essential for patient decision-making, treatment selection, and adjuvant therapy”[4]. To better guide treatment decisions, a need persists to more accurately characterize a patient's risk of metastasis.

Assessment of risk when considering postoperative secondary therapy is currently conducted based on individual clinicopathologic (clinical) variables and/or through use of nomograms [11]. However, the ability of these clinical variables to identify patients at substantially higher risk of metastasis and lethal prostate cancer is limited. Genomic features in the primary tumor reflect the true biological potential for disease progression and metastasis. Novel risk prediction tools that use such features can therefore provide the direct measure of risk that is needed. One such tool is a postoperative genomic classifier (GC) test (Decipher™, GenomeDx Biosciences, San Diego, CA) that uses a whole-transcriptome microarray assay for analysis of gene activity in formalin-fixed paraffin embedded prostate cancer specimens. Developed in collaboration with the Mayo Clinic, this GC was designed to predict early clinical metastasis following RP [12]. In a blinded clinical validation study of a contemporary high-risk population of post-RP men with adverse pathology, the GC test was found to more accurately predict metastasis post-RP than individual clinical variables, combinations of clinical variables or currently used nomograms [13, 14].

In assessing a novel molecular test, experts have recommended that evidence be collected not only on the clinical validity of the test, but also on how use of the test influences clinical practice management, a well-established measure of the test's clinical utility [15, 16]. The primary objective of this study was to determine how urologists' knowledge of the GC test results influenced adjuvant and salvage treatment recommendations following RP.

## RESULTS

Participating physicians were all practicing, high surgical volume urologists performing an average of 184 RPs per year (Table 1). Twenty-one urologists from 18 different institutions across the US participated: 20 in the adjuvant, and 15 in the salvage settings. Fourteen of these urologists completed assessment of cases in both sub-studies. Of the 21 urologists, 38% (n = 8) practice in a community-based hospital or private practice setting and 62% (n = 13) practice in tertiary care centers, the majority (85%) of which are National Cancer Institute (NCI) designated comprehensive cancer centers. Urologists had been practicing and performing surgery for 3 to 25 years (mean 8.1 years) and all have extensive experience managing and treating patients with prostate cancer both before and after RP.

**Table 1:** Characteristics of urologists participating in study

	Total	Adjuvant Evaluation	Salvage Evaluation
	n=21	n=20	n=15
	No. (%)	No. (%)	No. (%)
Practice setting			
Tertiary Care	13 (62%)	12 (60%)	9 (60%)
Community (hospital or private)	8 (38%)	8 (40%)	6 (40%)
Number of years in practice			
Mean	8.1	8.3	7.8
Range	3-25	3-25	3-25
Number of Radical Prostatectomy per year			
Mean	184	179	200
Range	30-300	30-300	30-300
Geographic region			
West/South Central	4 (20%)	4 (20%)	3 (20%)
South East	4 (20%)	4 (20%)	3 (20%)
Mid Atlantic	4 (20%)	3 (15%)	2 (13%)
North East	5 (25%)	5 (25%)	5 (33%)
North Central	4 (20%)	4 (20%)	2 (13%)

Characteristics of the twelve patients in each of the adjuvant and salvage settings are provided in Table 2. Half of the adjuvant patient cases were pre-operatively deemed low to intermediate risk according to D'Amico risk groups but were all subsequently up-graded/staged postoperatively. Furthermore, 75% of these cases presented with a pathologic Gleason score ≥7, and 36% were ≥65 years of age at the time of surgery. For cases reviewed in the salvage setting, 75% had a time to BCR ≤36 months, and 75% presented with a rapid PSAdt (<9 months). The majority (58%) of these cases were ≥65 years of age at the time of BCR.

**Table 2:** Demographic and clinical characteristics of patient cases in the adjuvant and salvage setting

	Adjuvant	Salvage
	No. (N=12) (%)	No. (N=12) (%)
Age (Years at RP or at BCR)		
Median (Min, Max)	60 (48, 70)	66 (57, 74)
Pre-operative Prostate-specific Antigen		
<10 ng/mL	10 (83.3)	9 (75)
10-20 ng/mL	1 (8.3)	2 (16.7)
>20 ng/mL	1 (8.3)	0
NA	0	1 (8.3)
D'Amico risk groups		
Low	2 (16.7)	1 (8.3)
Intermediate	4 (33.3)	7 (58.3)
High	6 (50)	4 (33.3)
Pathological Stage		
pT2N0M0	6 (50)	8 (66.7)
pT3N0M0	6 (50)	4 (33.3)
Extracapsular Extension		
Present	5 (41.7)	3 (25)
Seminal Vesicle Invasion		
Present	4 (33.3)	2 (16.7)
Surgical Margin Status		
Positive	8 (66.7)	6 (50)
Pathological Gleason Score		
6	3 (25)	0
7 (3+4)	4 (33.3)	2 (16.7)
7 (4+3)	1 (8.3)	4 (33.3)
8	1 (8.3)	5 (41.7)
9	2 (16.7)	1 (8.3)
10	1 (8.3)	0
Time to BCR (months)		
Median (Min, Max)	NA	16 (1, 112)
≤36 months	NA	9 (75)
>36 months	NA	3 (25)
PSAdt		
<6 months	NA	5 (41.7)
≥6 months	NA	6 (50)
<9 months	NA	9 (75)
≥9 months	NA	2 (16.7)
NA	NA	1 (8.3)

Abbreviations: RP, radical prostatectomy; BCR, biochemical recurrence; PSAdt: PSA doubling time

**Figure 1:**
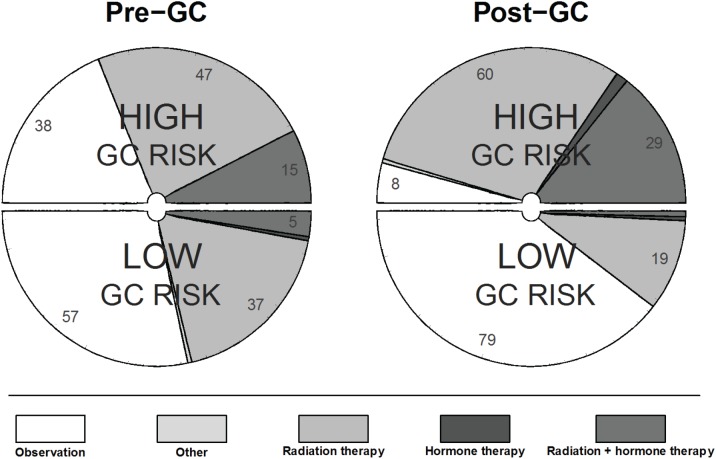
Breakdown of treatment recommendations pre-GC and post-GC for low and high GC risk groups in the adjuvant setting.

In the adjuvant treatment setting, 43% (95% CI: 37-49%) of recommendations changed following review of the GC test results (Table 3). Specifically, among case evaluations with a pre-GC recommendation involving treatment, 27% (95% CI: 19-35%) of recommendations changed to observation post-GC. Notably, for case evaluations with a pre-GC recommendation of radiation alone (n=100), 31% (95% CI: 22-41%) changed to observation post-GC (Table 3). Among the case evaluations where observation was initially chosen (n=114), treatment was recommended for 37% of case evaluations post-GC, primarily in favor of radiation therapy alone (37/42). This can be visualized in Figure 1, which shows how in comparison to pre-GC, post-GC urologists' recommendations for observation or treatment (radiation and/or hormones) aligned to a high degree with the risk assigned by the GC test. Detailed results for all possible combinations of pre- and post-GC treatment recommendations are provided in Supplementary Table S1.

**Table 3:** Effect of the GC test result on urologists treatment recommendations post radical prostatectomy

Treatment Recommendation		Adjuvant			Salvage		
Pre-GC	Post-GC	Pre-GC	Change N (%)	95% CI	Pre-GC	Change N (%)	95% CI
Overall	Any Change	240	103 (43%)	37-49%	180	95 (53%)	45-60%
Observation	Any Treatment	114	42 (37%)	28-46%	31	19 (61%)	42-78%
	Radiation	114	37 (32%)	24-42%	31	12 (39%)	22-58%
	Hormone therapy	114	4 (4%)	1-9%	31	0 (0%)	
	Radiation + Hormone therapy	114	1 (0.9%)	0-5%	31	7 (23%)	10-41%
	Other*	114	1 (1%)	0-5%	31	2 (7%)	0.8-21%
Any Treatment	Observation	125	34 (27%)	19-35%	143	23 (16%)	11-23%
Radiation	Observation	100	31 (31%)	22-41%	82	11 (13%)	7-23%
Hormone therapy	Observation	1	1 (100%)	3-100%	6	1 (17%)	0.4-64%
Radiation + Hormone therapy	Observation	24	2 (8%)	1-27%	55	11 (20%)	10-33%
Other*	Observation	1	1 (100%)	3-100%	6	0 (0%)	

* In the advjuant setting, ‘other’ treatment recommendations included: “recheck path” and “medical oncologist and radiation oncologist consult”

* In the salvage setting ‘other’ treatment recommendations included: “DRE, imaging” x3, “DRE, imaging, possible referral to radiation oncologist” x2, and “referral to medical oncologist”

Pre-GC treatment recommendations shown on left, post-GC treatment recommendation shown on right. Numbers indicate the % of patient cases for which a specific treatment recommendation was made in the respective GC group (100% for each GC risk group).

In the salvage setting, treatment recommendations changed 53% (95% CI: 45-60%) of the time (Table 3). Among case evaluations with a pre-GC recommendation involving treatment (n=143), 16% (95% CI: 11-23%) changed to observation post-GC. Expectedly, there were fewer pre-GC recommendations of observation (n=31) for case evaluations with BCR. Of these, 61% (n=19) were recommended to change from observation to involve treatment post-GC with radiation alone (n=12) or in combination with hormonal therapy (n=7) (Table 3). Similar to the analysis of the adjuvant setting above, we observe a trend that shows alignment of observation versus treatment recommendations with the risk assigned by the GC test, even though treatment recommendation rates were higher overall in the salvage setting (Figure S1).

Results were virtually unchanged when the possible correlation of recommendations from the same urologist (i.e. intra-observer correlation) were accounted for (refer to Supplementary Material).

To further examine the impact of the relationships between clinical variables and the GC test results on urologists' treatment recommendations, we evaluated the proportion of urologists recommending treatment pre- and post-GC over the complete set of case evaluations, as well as within individual clinical variables for high and low GC risk patients (Table S2). GC risk was established based on whether the predicted probability of developing metastasis was above (high GC risk) or below (low GC risk) the average risk for the original study population (see methods). Overall, in the adjuvant setting, treatment was recommended 52% of the time pre-GC. Post-GC, those with a low GC risk were recommended treatment only 21% of the time (i.e., 79% recommended to observation) compared to those with a high GC risk who were recommended treatment 90% of the time (p < 0.0001). Similarly, in the salvage setting, the overall proportion of treatment recommendation was 79% pre-GC, but post-GC fell to 75% in the low GC risk group and rose to 85% in the high-risk GC group (p = 0.031).

**Figure 2:**
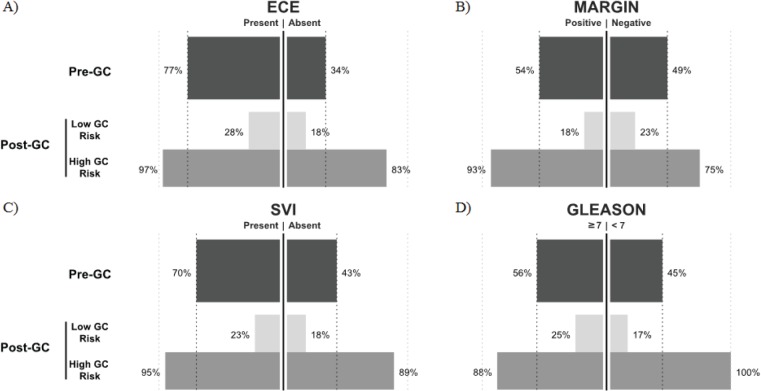
Proportion of recommendations for treatment for the indicated values of clinical variables (e.g. Presence/Absence) Pre-GC and the resulting proportion recommended for treatment post-GC in high and low GC risk groups in the adjuvant setting.

When evaluating individual clinical variables in the adjuvant setting (Table S2), cases with ECE present had the highest marginal rate of treatment recommendation pre-GC (77%); this fell to 28% for low GC risk case evaluations and rose to 97% for high GC risk case evaluations post-GC (p < 0.0001) (Figure 2). Similarly, in cases with positive surgical margins, 54% were recommended treatment pre-GC. Treatment recommendation dropped to 18% for cases with low GC risk and rose to 93% in high GC risk cases (p < 0.0001). For cases with pathological Gleason score ≥7 disease, 65% were recommended treatment pre-GC; among those with low GC risk only 25% were recommended treatment versus 90% of those cases with high GC risk (p < 0.01). The largest magnitude in change was observed in cases with SVI. Pre-GC, 70% of SVI cases were recommended treatment, but among those cases with low GC risk, only 23% were recommended treatment in the presence of SVI. In high GC risk cases with SVI, 95% were recommended for treatment (p < 0.0001). These results reinforce the impact of the GC test and indicate that the rate of treatment may be strongly associated with the GC risk (or probability of developing metastasis) than any other clinical variable (Table S2, Figure 2).

A) Extracapsular Extension (ECE), B) Surgical Margins, C) Seminal Vesicle Invasion (SVI) and D) Gleason score. Proportions of patients recommended treatment indicated on x-axis (%) depending on clinical variable status for pre-GC (dark grey, top), low GC risk (lightest grey, middle) and high GC risk (light grey, bottom). High GC tends to result in treatment rates higher than pre-GC while low GC tends to result in treatment rates lower than pre-GC.

In the salvage setting, pre-GC the main drivers for recommendation of treatment were PSAdt and time to BCR. Cases with a rapid PSAdt of <6 months were recommended for treatment by 93% of urologists pre-GC. However, the proportion dropped to 73% within low GC risk patients, post-GC. For cases with longer PSAdt (and hence a presumed better prognosis), only 14 (47%) recommendations for treatment were made pre-GC, but this increased to 25 (83%), post-GC, and all of these cases had high GC risk. As in the adjuvant setting, presence or absence of individual clinical variables (except for margin status) did not influence recommendations to treat post-GC; in this setting, GC risk was the main driver (Table S2, Figure S2).

Results for univariable and multivariable regression models for recommendation of treatment pre- and post-GC are included in Table S3. In the adjuvant setting, clinical variables influence urologists' recommendation to treat pre-GC. When the urologists are aware of the genetic risk, post-GC, only GC risk (p < 0.0001) and ECE (p = 0.018) have a significant impact on the recommendation for treatment. While not significant in the salvage setting, GC risk has the lowest p-value of all variables in the post-GC multivariable model. Since cases were not randomly sampled from the general population, these results are considered exploratory.

To measure recommended changes in treatment intensity, we established a baseline clinical perception of risk (hereafter referred to as perceived risk). Cases were considered low perceived risk if less than half of urologists recommended treatment and high perceived risk if more than half recommended treatment in the absence of the GC test results. In the adjuvant and salvage settings we observed that if perceived risk was high but GC risk was low, then, respectively, 50% and 46% of recommendations reduced treatment intensity post-GC (e.g., radiation to observation or radiation/hormone combination to radiation only) (Table 4). Very few recommendations were made that increased treatment intensity, (only 5% and 3.8%, respectively for adjuvant and salvage treatment recommendations). Conversely, for cases with an initial low perceived risk but high GC risk, we observed a 55% and 58% increase in treatment intensity in both the adjuvant and salvage settings, respectively. Influence of GC risk on change in intensity for all clinical risk categories and treatment settings were highly statistically significant (p < 0.0001). Furthermore, a multivariable model adjusting for the pre-GC clinical risk showed that GC risk influenced change in treatment recommendation intensity (p < 0.0001).

**Table 4:** Change in treatment intensity from initial perceived clinical risk compared against GC risk.

Timepoint	Perceived Risk	GC Risk	Decrease	No Change	Increase
Adjuvant	high	low	20 (50%)	18 (45%)	2 (5%)
		high	3 (5%)	35 (58.3%)	22 (36.7%)
	low	low	15 (18.8%)	60 (75%)	5 (6.3%)
		high	3 (5%)	24 (40%)	33 (55%)
Salvage	high	low	48 (45.7%)	53 (50.5%)	4 (3.8%)
		high	1 (3.3%)	17 (56.7%)	12 (40%)
	low	high	4 (8.9%)	15 (33.3%)	26 (57.8%)

Low (High) Perceived Risk = < half (> half) of clinicians initially recommend treatment

Low (High) GC Risk at Advjuvant timepoint = 5 year predicted probability < 6% (> 6%)

Low (High) GC Risk at Salvage timepoint = 3 year predicted probability < 18% (> 18%)

To understand the extent to which the GC test result impacts confidence in making a treatment recommendation, urologists were asked to report on the degree to which they felt confident in the treatment recommendation made for case evaluations both pre- and post-GC, as well as, the extent to which they felt the GC test result influenced those treatment recommendations. Results showed that for case evaluations where a treatment recommendation was made, urologists' confidence in treatment recommendations increased by 25% and 23% after reviewing the GC test result in the adjuvant and salvage settings, respectively. Additionally, urologists reported that the GC test result influenced their treatment recommendation in 84% (adjuvant) and 87% (salvage) of case evaluations (Table S4).

## DISCUSSION

This clinical utility study was designed to prospectively assess the effect of a genomic classifier (GC) test that predicts metastasis following RP on urologists' adjuvant and salvage treatment recommendations. The performance of the GC test was previously reported in a blinded, independent validation study of a population of 1,010 men at high risk of recurrence (based on adverse pathology) post-RP [14]. That study revealed that 60% of clinically high-risk patients would be reclassified as low-risk with a cumulative incidence of metastasis of only 2.4% at 5 years post-RP. Conversely, patients with the highest GC scores (19% of the cohort) had nearly 10 times higher cumulative incidence of metastasis by 5 years. Findings from this current study demonstrate that knowledge of the GC test result frequently impacted urologists' treatment recommendations in both the adjuvant (43%) and salvage settings (53%). Furthermore, in the adjuvant setting we were able to show that for patients with low GC risk, while pre-GC urologists recommended treatment 43% of the time, post-GC they were recommended to observation 79% of the time (Figure 1). Taken together, the clinical validation and utility results imply that among the population of prostate cancer patients at high-risk of recurrence following RP, the majority of patients tested will be recommended to close observation post-GC thereby sparing them the morbidity and costs associated with secondary therapy post-RP.

Guidelines on evidence development for molecular tests drafted in the past 3-5 years have urged going beyond obtaining evidence on an assay's analytical and clinical validity, encouraging additional research on how a test influences clinical practice management [15], [18]. To date in this nascent field, the number of published studies is fairly limited, but growing. In a clinical study assessing a molecular assay for stage II colon cancer, Srivastava et al. found that physicians changed chemotherapy decisions in 45% of patients [19], which fully validated predictions from a simulation of changes in NCCN guideline-directed treatment [20]. One of the most studied areas of practice management change in molecular medicine has been risk prediction in breast cancer. In a comprehensive and systematic review of clinical validity and changes in clinical practice patterns, Hornberger et al. found 15 studies reporting on 5 different tests [21]. They found that chemotherapy recommendation changed between <1%-13% as reported in 4 studies of an online clinical decision support tool, compared with a median change across all studies of less than 35% in recommendations for a multi-gene assay. In comparison with these examples of accepted oncology tests, the finding in our study of a 43-53% change in recommendation upon receipt of the test results is supportive evidence that the GC test provides additional useful information to guide therapy selection

This study reveals relevant findings relating to current practice patterns for high-risk prostate cancer patients post-RP and confirms urologists' proclivity for not only increased salvage treatment at the point of BCR but also increased intensification of treatment when compared to the adjuvant setting. Overall, urologists recommend treatment over 1.5 times as often in the salvage versus the adjuvant setting; treatment recommendations were made for 79% of case evaluations pre-GC in the salvage setting, 39% of which involved a recommendation for multi-modal (i.e., radiation and hormone) therapy. This compares to a recommendation for multi-modal therapy in only 19% of case evaluations pre-GC in the adjuvant setting. In addition, the findings imply a potential to over-treat in the salvage setting as evidence suggests that even in patients presenting with BCR, less than one-third will go on to develop metastasis [5]. This is not without consequences for the patient and the healthcare system as both postoperative radiation and hormone therapy incur with considerable costs and morbidities including bladder and rectal complications as well as urinary incontinence and impotence, which can affect both near and long-term patient quality of life [22].

Results from this study also confirm that urologist decision-making in the adjuvant setting is mainly focused on whether or not to recommend postoperative radiation therapy. Prior to presentation of the GC test results, urologists recommend treatment in 52% of case evaluations with 99% of those recommendations including radiation therapy and only 20% of recommendations including hormone therapy. Accurate direction of radiation therapy to patients who are at highest biological risk for developing metastasis is critical as the morbidities and costs associated with treating patients with radiation modalities such as IMRT run high [23]. Furthermore, we observed that the GC risk significantly influenced the treatment recommendations irrespective of the presence or absence of specific clinical variables. Additionally, these findings confirm the widely accepted observation that in the salvage setting, the sensitivity of PSA rise may motivate urologists to recommend treatment despite its poor specificity. This hints towards a role for the GC test to improve urologist decision-making in this setting. Similar results were found relating to the intensification of treatment, where changes in intensity were driven primarily by GC risk rather than the perceived risk. This suggests that given the information from the GC test, presumably measuring the true biological potential of a patient's tumor, urologists are more willing to commit to the intensification of therapy than if this recommendation were solely based on rising PSA and clinical variables (i.e., pre-GC).

The results of this study provide insights to the potential role of the GC test despite the limitations of the study. Paramount among these is the non-random selection of cases and the fact that they do not represent the expected distribution of perceived or GC risk in the population at large. In particular, perceived and GC high-risk patients are over-represented in this study. Low GC risk cases tended to result in less treatment, but a larger study to more accurately estimate the influence in this substantial element of the population is necessary. Furthermore, the small number of physicians involved may not represent urologists at large; although accounting for intra-physician correlation (refer to Supplementary Material) resulted in a negligible impact on the overall probability of recommendation change. Additional studies are planned to include a larger random sample of both patient cases and urologists.

To our knowledge this is the first study to assess the effect of a molecular/genomic test on physician treatment recommendations in prostate cancer. Treatment recommendations changed in 43% of adjuvant setting case evaluations and 53% of salvage setting case evaluations. These findings demonstrate that knowledge of the genomic biomarker information in this GC test frequently influences these urologists' judgments about appropriate secondary therapy in both the adjuvant and salvage settings. These estimates are encouraging, but exclude near- and long-term implications on improved patient quality of life through more targeted recommendation of therapies, change in adverse event rates with secondary therapies such as radiation therapy, or direct costs associated with these therapies. Additionally, cost implications of supportive care, management of adverse events, delaying progression, or end-of-life care may provide a more comprehensive view of the true impact of novel genomic tests. As recommended in guidelines, it will be important to combine the findings of this study with other data to assess the E (i.e. ethical, legal and social implications) in the ACCE criteria.

## MATERIALS AND METHODS

This clinical utility study used a prospective, pre-post design, consisting of two independent sub-studies to assess patient cases at different points in patient management; both are collectively referred to herein as the DECIDE study. In one sub-study, urologists' treatment recommendations were assessed in the adjuvant setting, following RP without any evidence of PSA rise/BCR. In the other, treatment recommendations were assessed for a different cohort of cases in the salvage setting, following RP with evidence of PSA rise/BCR. Urologists were invited to review de-identified clinical variables on a set of twelve real patient cases in each sub-study and provide treatment recommendations. Cases were obtained from a previous clinical validation study [13, 14]. In both the adjuvant and salvage sub-studies, recommendations were first obtained based solely on the clinical variables provided (pre-GC). Then, results of the GC test were assessed for the same de-identified cases and urologists were asked again to provide treatment recommendations (post-GC). Twenty urologists participated in the adjuvant setting study and 15 in the salvage setting study (Table 1).

The study was conducted in accordance with the Declaration of Helsinki and the Belmont report and was reviewed and approved by an independent IRB (Quorum Review Inc., Seattle, WA).

The primary objective of this study is to assess the effect of the GC test on urologists' adjuvant and salvage treatment recommendations for clinically and pathologically high-risk post-RP cases. Secondary objectives were to investigate specific changes in recommendations such as, proclivity of the GC test to result in more or less intensification of treatment, the relative importance of the GC to clinical variables and impact of the GC on urologists' confidence with treatment recommendations. Protocol-defined eligibility criteria for participation in the study required US board certified urologists practicing for at least 3 years and performing a high volume of RPs annually (Table 1). All urologists participating in the study were fellowship trained urologic oncologists. Eligible participants were identified through conference delegate lists and through established networks of key opinion leaders. Email invites were sent to 50 urologists meeting the inclusion criteria. Enrollment packages were sent to eligible urologists interested in participating in the study and included a cover letter, an educational primer on the GC test, a confidentiality agreement and a web link to the study's informed consent form (ICF) and electronic case report questionnaires (eCRQ).

Twenty-four high-risk, post-RP patient cases (12 adjuvant and 12 salvage) were selected for urologist review from the previously conducted clinical validation study [13, 14]. The number of patient cases was selected to provide enough cases to adequately evaluate urologists' decision making across a range of high-risk patient types and was limited to twelve cases in each treatment setting so as to minimize study participant fatigue in reviewing cases. All cases were high-risk, post-RP as defined by the presence of one or more adverse pathological features including (1) pathological Gleason score 8+ or Gleason score 7 with primary pattern 4; (2) pathological stage T3a (ECE) or T3b (SVI); (3) positive surgical margins; or (4) Gleason grade upgrade from biopsy to RP. Selected cases did experience PSA nadir after RP.

These high-risk cases were further selected for inclusion in the study on the basis of the GC predicted probability of developing metastatic disease at 5 years post-RP and 3 years post-BCR for the adjuvant and salvage treatment settings, respectively. High (low) GC risk was defined as a 5- or 3-year predicted probability of metastasis greater (less) than 6% for the adjuvant setting and greater (less) than 18% for the salvage setting. In the adjuvant setting, six cases with low GC risk and six cases with high GC risk were selected. In the salvage setting, these numbers were 5 and 7, respectively. Clinical assessment for each patient case was presented based on the following variables: age at surgery, pre-operative PSA levels, pathologic stage, biopsy and pathologic Gleason score, presence or absence of SVI and ECE, surgical margin status and lymph node involvement (Table 2). Additionally, PSAdt and time to BCR were provided for cases evaluated in the salvage setting. These thresholds were based on the average risk of metastasis in the original study population [13, 17]. Actual patient outcomes (i.e.: progression status at any time point) were not presented for assessment.

All cases were de-identified and presented in a randomized fashion to eliminate bias toward the urologist's pre- and post-GC treatment recommendations. Cases were randomized both from urologist to urologist and from pre- to post-GC. Clinical variables and GC test result information were provided to urologists through a secure online platform, and all treatment recommendations were collected using the eCRQ. Treatment recommendations included referral to a radiation oncologist and/or initiation of hormones, close observation, or any other recommendation not listed on the eCRQ.

Confidence intervals for probability of recommendation change from pre- to post-GC were constructed using a normal approximation, a significance level of 5%, and all recommendations were considered as independent. Chi-squared tests were used for univariate assessment of treatment predictors and multivariable analyses were performed using logistic regression. All statistical analyses were performed using SAS 9.2 (Cary, NC). All tests were 2-sided with a Type I error probability of 5%.

## CONCLUSION

The DECIDE study assessed the effect of the GC test on urologist treatment recommendations for high-risk case evaluations in the adjuvant and salvage treatment settings post-RP. Findings demonstrate that knowledge of the GC test result frequently impacted urologists' treatment recommendations in both the adjuvant and salvage settings. Furthermore, the GC test appears to better direct urologist treatment recommendations irrespective of the presence or absence of conventional pathology and clinical variables that are currently used to assess risk in these patients.

In conclusion, this study suggests that when implemented into routine clinical practice, the GC test has the potential to change treatment recommendations after radical prostatectomy and better identify patients that may benefit from intensive multimodal therapy, while sparing those who can be closely observed without initiating aggressive secondary therapy and in doing so, holds the potential to improve patient outcomes, decrease morbidities and ultimately reduce costs to the healthcare system.

## Supplementary Figures and Tables




