# Supplementary material for: Impact of a genomic classifier of metastatic risk on postoperative treatment recommendations for prostate cancer patients: a report from the DECIDE study group
Source: Oncotarget. 2013 Sep 8;4(4):600–9. doi: 10.18632/oncotarget.918 (PMC3720607; doi:10.18632/oncotarget.918)
Supplement: Supplementary file 1 [file oncotarget-04-600-s001.pdf]

Impact of a genomic classifier of metastatic risk on postoperative treatment recommendations for prostate cancer patients: a report from the DECIDE study group - Badani et al

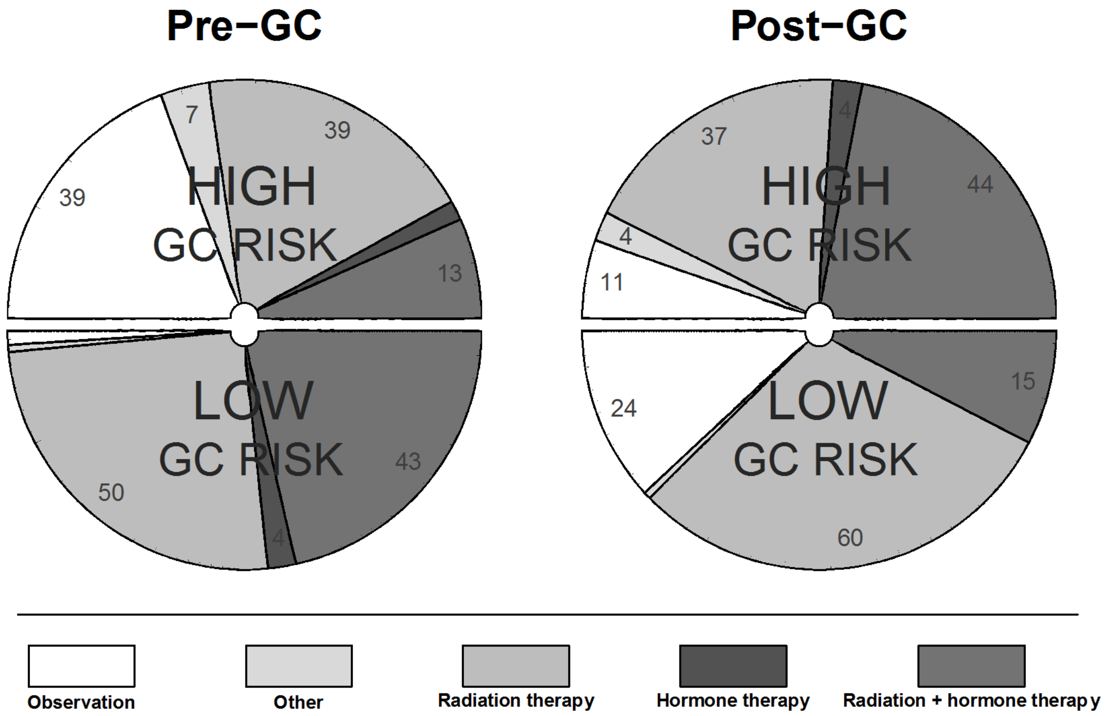

Figure S1: Breakdown of treatment recommendations pre-GC and post-GC for low and high GC risk groups in the salvage setting. Pre-GC treatment recommendations shown on left, post-GC treatment recommendation shown on right. Numbers indicate the % of patient cases for which a specific treatment recommendation was made in the respective GC group (100% for each GC risk group). Treatment patterns change substantially from pre-to post-GC with the proportion of recommendations for observation increasing considerably in the low GC risk group (2% to 24%) and decreasing the high GC risk group (39% to 11%).

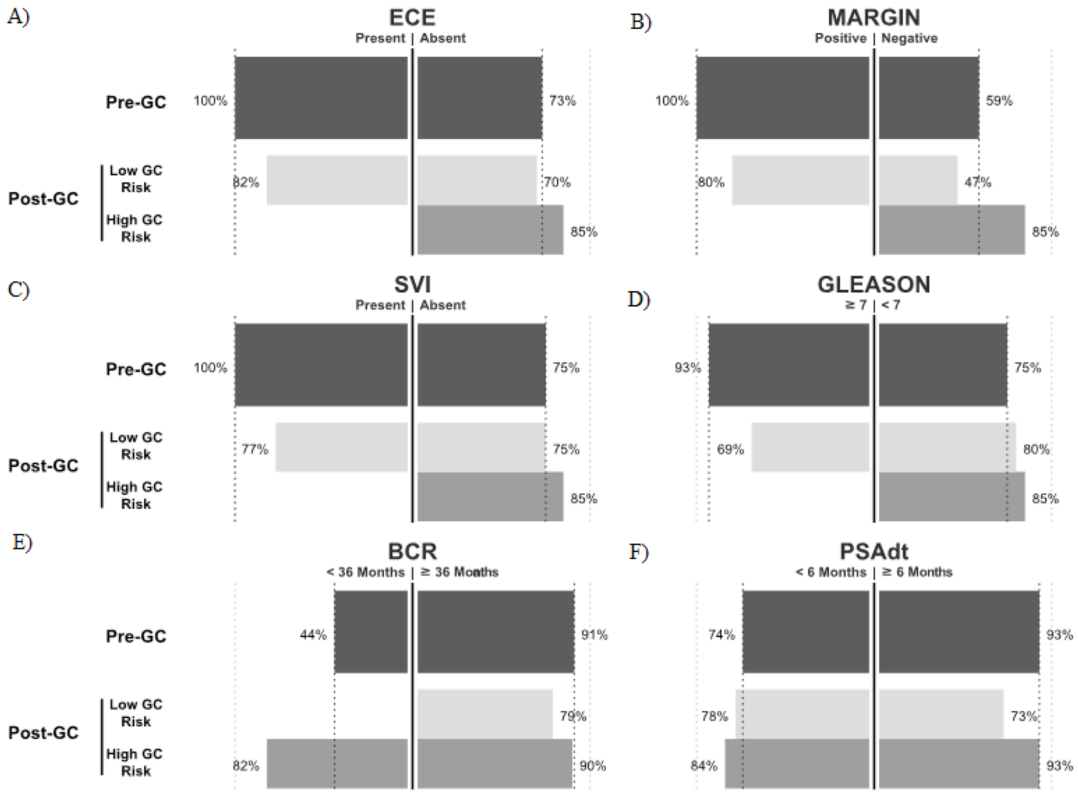

Figure S2: Proportion of recommendations for treatment for the indicated values of clinical variables (e.g. Presence/ Absence) Pre-GC and the resulting proportion recommended for treatment post-GC in high and low GC risk groups in the salvage setting. A) Extracapsular Extension (ECE), B) Surgical Margins, C) Seminal Vesicle Invasion (SVI), D) Gleason score, E) Time to BCR and F) PSA doubling time (PSAdt). Proportions of patients recommended treatment indicated on x-axis (%) depending on clinical variable status for pre-GC (dark grey, top), low GC risk (lightest grey, middle) and high GC risk (light grey, bottom). High GC risk tends to result in treatment rates as high or higher than pre-GC while low GC tends to result in treatment rates the same or lower than pre-GC.

Intra-observer correlation

Confidence intervals accounting for the correlation of multiple recommendations made by the same urologist (i.e.; intra-observer correlation) were constructed using a Generalized Linear Mixed Effects Model (GLMM) with physician as a cluster (PROC GLIMMIX in SAS).

When accounting for intra-observer correlation, the results are consistent with the independent recommendation analysis, with estimated probabilities of recommendation change of 43% (95% CI 36-50%) in the adjuvant setting and 53% (95% CI 39-67%) in the salvage setting.
